# Skim Milk Culture of *Lactobacillus johnsonii* SBT0309 Increases Intestinal Alkaline Phosphatase Activity and Inhibits Lipopolysaccharide-Induced Interleukin-8 Production in Intestinal Epithelial Cells

**DOI:** 10.3390/cells14050358

**Published:** 2025-02-28

**Authors:** Michio Kawano, Toshinobu Arai, Toshihide Kabuki

**Affiliations:** Milk Science Research Institute, MEGMILK SNOW BRAND Co., Ltd., 1-1-2 Minamidai, Kawagoe-shi, Saitama 350-1165, Japan

**Keywords:** fermented milk, skim milk culture, *Lactobacillus johnsonii* SBT0309, intestinal alkaline phosphatase, *Drosophila melanogaster*, Caco-2 cells, lipopolysaccharide

## Abstract

Background/Objectives: Intestinal alkaline phosphatase (IAP) is an enzyme expressed in the intestinal brush border, which may exert anti-inflammatory effects by detoxifying lipopolysaccharides (LPSs), thereby preventing metabolic disorders. Various food components have been reported to influence IAP activity. However, few studies have evaluated the effects of fermented milk on IAP activity. In this study, we aimed to investigate fermented milk with high IAP-activating capacity and investigate its effect. Methods: We screened a skim milk culture (SC), a fermented milk model, using differentiated Caco-2 cells. We investigated the effect of SC on IAP activity and gene expression in the *Drosophila* midgut. Quantitative PCR and immunoblot assays were conducted to examine gene and protein levels. Results: Among the SC samples from different lactic acid bacteria or bifidobacteria, the SC of *Lactobacillus johnsonii* SBT0309 (LJ0309 SC) demonstrated a particularly strong capacity to activate IAP in Caco-2 cells, demonstrated by significantly increased IAP gene expression and protein levels in Caco-2 cells. Additionally, LJ0309 SC inhibited increased secretion of IL-8 in LPS-stimulated Caco-2 cells. Finally, in *Drosophila melanogaster* fed LJ0309 SC, we observed an increase in both IAP activity and gene expression in the midgut. Conclusions: LJ0309 SC increased IAP activity and gene expression in both Caco-2 cells and the *Drosophila* midgut, and inhibited the inflammatory response in LPS-stimulated Caco-2 cells. Although further in vivo studies are required, LJ0309 SC might help to ameliorate LPS-induced inflammation and disease via IAP activation.

## 1. Introduction

The enzyme alkaline phosphatase (EC 3.1.3.1) is widely distributed in various organs, including the liver, bone, kidney, intestine, and placenta. When these organs are damaged, levels of alkaline phosphatase in the bloodstream are upregulated. Therefore, alkaline phosphatase is a known biomarker for the diagnosis of liver-, bone-, and other organ-related diseases [1].

Alkaline phosphatase is also a beneficial agent for health. There are four different isozymes of alkaline phosphatase in humans: tissue-nonspecific, placental, germ-cell, and intestinal alkaline phosphatase (IAP). Among these, IAP has been the most studied owing to its effects on health [2]. IAP, which is expressed at the intestinal brush border, contributes to the maintenance of intestinal homeostasis. Malo et al. showed that IAP-deficient mice have fewer and different types of aerobic and anaerobic bacteria in their stools than wild-type mice [3]. The administration of calf IAP (cIAP) to mice inhibits infection with pathogenic bacteria, such as *Salmonella enterica* serovar Typhimurium and *Clostridium difficile* [4]. Tuin et al. found that *IAP* gene expression was significantly lower in patients with inflammatory bowel diseases (IBDs), including Crohn’s disease and ulcerative colitis, and cIAP treatment prevented colonic inflammation in a dextran sulfate sodium-induced rodent model of IBD [5]. These results suggest that IAP exerts intestinal homeostatic effects by improving the gut microbiota and suppressing gut inflammation.

The function of IAP in maintaining intestinal homeostasis may be related to the dephosphorylating effect of IAP on lipopolysaccharide (LPS). LPS is a component of the cell walls of Gram-negative bacteria. It is a pro-inflammatory factor present in high levels in the gut. LPS is a toll-like receptor (TLR) 4 agonist that stimulates inflammatory signals such as those from the nuclear factor-kappa B (NF-κB) pathway and induces the secretion of the inflammatory cytokines tumor necrosis factor (TNF)-α, interleukin (IL)-1β, IL-6, and IL-8 [6]. Lipid A, the active component of LPS, contains monosubstituted phosphate or pyrophosphate groups in the glycosidic position [7]. Once IAP reacts with LPS, it releases a phosphate from lipid A and attenuates the inflammatory potential of LPS [8]. Compared with intact LPS, IAP-treated LPS induces lower levels of inflammatory cytokines in THP-1 monocyte cells [9]. Therefore, IAP might maintain intestinal homeostasis by dephosphorylating LPS, thereby reducing gut toxicity and inflammation.

IAP may have beneficial effects on bowel inflammation. LPS may cause systemic inflammation. Cani et al. reported that disruption of the intestinal barrier leads to the translocation of LPS from the gut to the blood and induces inflammation in various tissues, such as the liver and adipose tissue, which may contribute to insulin resistance and the development of metabolic syndrome [10]. As mentioned above, IAP attenuates LPS activity and maintains intestinal homeostasis, suggesting that it may inhibit systemic inflammation caused by LPS exposure. IAP-KO mice showed intestinal barrier disruption, increased LPS influx, progression of systemic inflammation, insulin resistance, and the development of liver damage; however, these symptoms were ameliorated in mice treated with cIAP [11,12]. Therefore, if potential IAP activity in the gut could be increased, it would be helpful in the prevention of insulin resistance and metabolic syndrome.

Various substances, including exogenous bacteria and dietary components, influence IAP activity [13]. For example, IAP is activated by *Escherichia* (*E.*) *coli* and its LPS [14,15], as well as by hormones [16], cytokines [17], fatty acids [18], and sugars [19]. However, L-phenylalanine [20] can inhibit its enzymatic activity. Exogenous bacteria and food components also affect IAP activity; therefore, IAP might be activated in fermented foods containing bacteria. Milk fermented by lactic acid bacteria can increase IAP activity, and has a more powerful effect than skim milk [21]. The identification of lactic acid bacteria with potent IAP activation potential could lead to the development of fermented milk products that increase IAP activity and help prevent metabolic diseases.

In this study, we carried out an in vitro screening of skim milk culture (SC), a model of fermented milk, to select strains of lactic acid bacteria or bifidobacteria that could be used to produce SC with high IAP activation capacity. We then investigated the effect of the SC of *Lactobacillus johnsonii* SBT0309 (LJ0309 SC), which was identified in the screening, on *IAP* gene expression and protein levels in intestinal epithelial cells. The effects of LJ0309 SC on LPS-stimulated intestinal epithelial cells were also investigated. Finally, *Drosophila melanogaster*, an alternative to animal testing, was used to test the ability of LJ0309 SC to activate IAP in the gut.

## 2. Materials and Methods

### 2.1. Materials

The Caco-2 cells were purchased from KAC (Kyoto, Japan). LPS derived from *E. coli* O55:B5 was purchased from Sigma-Aldrich (St. Louis, MO, USA). Dulbecco’s modified Eagle’s medium (DMEM), nonessential amino acid solution (NEAA), and sodium butyrate were purchased from Nacalai Tesque (Kyoto, Japan). Fetal bovine serum (FBS), penicillin–streptomycin solution (P/S), and M17 broth were purchased from Thermo Fisher Scientific (Waltham, MA, USA). De Man, Rogosa, and Sharpe broths were purchased from BD Biosciences (Franklin Lakes, NJ, USA). Gifu Anaerobic Medium was purchased from Nissui Pharmaceutical (Tokyo, Japan). Meast powder N (yeast extract) and HB-P02 (beer yeast) were purchased from Asahi Group Food (Tokyo, Japan). Skim milk was obtained from the MEGMILK SNOW BRAND (Sapporo, Hokkaido, Japan). Phenylmethylsulphonyl fluoride (PMSF), propionic acid, and methyl 4-hydroxybenzoate were purchased from Fujifilm Wako Pure Chemicals (Osaka, Japan). Bacterial alkaline phosphatase (BAP) was purchased from Takara Bio (Kusatsu, Shiga, Japan). Corn flour was purchased from the NIPPN CORPORATION (Tokyo, Japan). The standard *Drosophila* strain *w*^1118^ (RRID: BDSC_3605) was obtained from the Bloomington Drosophila Stock Center (Indiana University, Bloomington, IN, USA).

### 2.2. Preparation of SC Samples

For screening experiments, we made a medium containing 10% skim milk, 0.5% Yeast powder N, 1% glucose, and 0.1% sodium ascorbate, and sterilized it in an autoclave at 115 °C for 20 min. Filter-sterilized 2% l-cysteine hydrochloride was added to the sterilized medium at a final concentration of 0.04% (SM).

The bacterial strains were cultured in the optimal culture medium (Tables S1 and S2) and subsequently cultured in SM for three transfers. After measuring the pH, the third-passage cultures were lyophilized and stored at −80 °C until use (lyophilized SC samples). Except for the screening experiments, LJ0309 was cultured in SM without glucose, sodium ascorbate, and L-cysteine hydrochloride, which are required for the growth of some bacteria, such as bifidobacteria, but not for LJ0309.

### 2.3. Cell Culture Experiments

Caco-2 cells were seeded at 0.5 × 10^5^ cells/well on collagen-coated 48-well plates (AGC Techno Glass, Shizuoka, Japan) and cultured in DMEM containing 10% FBS, 1× NEAA, and 1× P/S for 2 weeks. Two weeks later, the cells were cultured in a cell culture medium containing different samples (5 mM sodium butyrate, 1–10 mg/mL lyophilized SM, or 1–10 mg/mL lyophilized SC samples) with or without 10 µg/mL LPS and incubated for 7 days. All media were refreshed every 2–3 days.

### 2.4. Drosophila Experiments

Flies were reared and maintained on a standard cornmeal/yeast diet (50 g/L glucose, 45 g/L beer yeast, 40 g/L corn flour, 8 g/L agar, 4 mL/L propionic acid, and 0.3 mL/L methyl 4-hydroxybenzoate) in a 12 h light/12 h dark cycle. Experimental diets were prepared by mixing 2.5% (*w*/*v*) lyophilized SM or 2.5% (*w*/*v*) lyophilized LJ0309 SC with a cornmeal/yeast diet. Three-day-old virgin female flies were transferred to vials containing the experimental diet (15–20 flies per vial). The flies were maintained for 5 weeks by changing the diet vials every 2–3 days. After the experimental period, flies were anesthetized using CO_2_, washed with 70% ethanol, and placed on a glass slide filled with TBS buffer containing 1 mM PMSF and 10 mM TBS (pH 7.3). The gastrointestinal tract was extracted from the body using forceps. The foregut, hindgut, and Malpighian tubes were then removed. The midgut was collected and used for subsequent assays.

### 2.5. IAP Activity Assay

Caco-2 cells were washed three times with ice-cold saline and lysed in lysis buffer containing 1% Triton X-100, 1 mM PMSF, and 10 mM TBS (pH 7.3). The lysed samples were sonicated on ice for 15 s at 30% amplitude using a SONIFIER SFX150HH (Branson Ultrasonics, Danbury, CT, USA). The sonicated samples were centrifuged at 4800× *g* for 5 min at 4 °C, and the supernatants were collected. The supernatants were used as crude cell extracts. In the *Drosophila* experiments, the three midgut samples were collected into a tube containing 100 µL of lysis buffer, homogenized with a pestle, and used as a crude extract of midgut.

To measure the IAP activity of the crude extracts, we used a Pierce PNPP Substrate Kit (Thermo Fisher Scientific). A calibration curve was constructed using BAP and the IAP activity of the samples was calculated. The protein concentration of the crude extract was measured using a Pierce BCA Protein Assay Kit (Thermo Fisher Scientific) and used to correct for IAP activity.

### 2.6. Cytokine Assays

Culture fluid from Caco-2 cells was centrifuged to remove debris and supernatants were stored at −80 °C. Thereafter, TNF-α, IL-1β, IL-6, IL-8, and IL-10 were determined using human ELISA MAX kits (BioLegend, San Diego, CA, USA).

### 2.7. Gene Expression Analysis

After three washes with ice-cold saline, we isolated total RNA from Caco-2 cells using Maxwell RSC simplyRNA Tissue Kits and a Maxwell RSC Instrument (Promega, Madison, WA, USA).

Total RNA extracted from five *Drosophila* pulverized midgut using an RNeasy Mini Kit (Qiagen, Hilden, Germany) was reverse-transcribed into cDNA using ReverTra Ace Master Mix (Toyobo, Osaka, Japan). Targeted cDNA was amplified using real-time quantitative polymerase chain reaction (qPCR) using THUNDERBIRD NextSYBR qPCR Mix (Toyobo) and a QuantStudio 6 Pro Real-Time PCR System (Thermo Fisher Scientific Inc.). The RNA extraction, cDNA synthesis, and qPCR proceeded as described by the manufacturers. The amplification conditions comprised 40 cycles of denaturation at 95 °C for 15 s, annealing at 60 °C for 30 s, and extension at 72 °C for 60 s. Relative gene expression was calculated from standard curves. The endogenous controls were *18S rRNA* and *Rp49* for the cell culture and *Drosophila* experiments, respectively. Table S3 shows the primer sequences.

### 2.8. Immunoblotting Analysis

After three washes with ice-cold saline, Caco-2 cells cultured in 48-well plates were lysed as described above. Debris was removed from the lysates by centrifugation; then, the supernatants in 4× sample buffer containing 10% 2-mercaptoethanol (Bio-Rad Laboratories Inc., Hercules, CA, USA) were heated at 95 °C for 10 min. Proteins were resolved with 12.5% SDS-PAGE (Bio-Rad Laboratories Inc.), then immunoblotted using anti-IAP (ab186422; Abcam, Cambridge, UK) and anti-β-actin (M177-3, MBL, Tokyo, Japan) antibodies. Blots were, respectively, visualized and quantified using ChemiDoc MP and Image Lab 4.1 software (both from Bio-Rad Laboratories Inc.).

### 2.9. Statistical Analysis

Data are expressed as means ± standard deviation (SD). Data were statistically analyzed using Statcel4 (OMS Publishing Co., Saitama, Japan) and EZR [22]. Between-group differences were analyzed using Student *t*-tests and multiple groups were compared using one-way ANOVA followed by Dunnett tests between controls and other groups. The effects of LJ0309 SC on mRNA expression and IL-8 secretion in Caco-2 cells stimulated with LPS were analyzed using two-way ANOVA, followed by Tukey–Kramer tests. Values with *p* < 0.05 were considered statistically significant. We did not apply non-parametric tests because all statistical analyses were parametric.

## 3. Results

### 3.1. Screening of SC to Increase IAP Activity of Caco-2 Cells

For the screening experiment, we used a Caco-2 cell monolayer model, which is commonly used in IAP studies [23]. We prepared SC samples using 128 strains of 55 bacterial species (Table S1) and screened them for those that showed increased IAP activity in Caco-2 cells. The 128 SC samples were divided into four groups (Groups 1–4), and the 3 SC samples with the highest IAP activation capacity were selected for each group (Figure S1). We then compared the IAP activation activity of the selected SC samples and found that *Lactobacillus delbrueckii* subsp. *jacobsenii* SBT0803, *L. johnsonii* SBT0307, and *Streptococcus thermophilus* SBT1016 are bacterial strains that can produce SC samples that enhance IAP activity in Caco-2 cells (Figure S2a). Although the SC of *Bifidobacterium mongoliense* SBT2491 also increased IAP activity in Caco-2 cells, the SC samples of the other three strains were able to increase IAP activity to a greater extent (Figure S2b). Therefore, it was suggested that *L. delbrueckii* subsp. *jacobsenii* SBT0803, *L. johnsonii* SBT0307, and *S. thermophilus* SBT1016 could produce SC with a relatively strong activating capacity for IAP.

**Figure 1:**
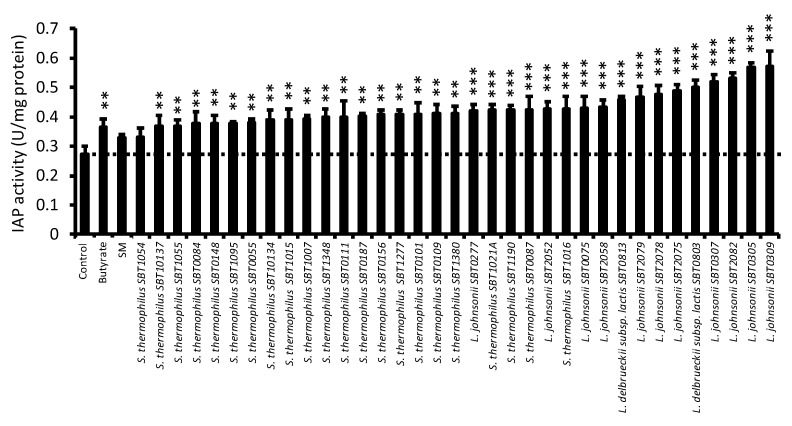
Screening of SC samples to increase IAP activity of Caco-2 cells. IAP activity in differentiated Caco-2 cells treated with butyrate (2 mM), lyophilized SM (5 mg/mL), or lyophilized SCs (5 mg/mL) for 7 days. SC samples were prepared with *Lactobacillus* (*L.*) *delbrueckii* subsp. *jacobsenii*, *L. johnsonii,* or *Streptococcus thermophilus* as listed in Table S2. Data are shown as mean + SD (n = 3) and analyzed using one-way ANOVA followed by Dunnett’s test (** *p* < 0.01, *** *p* < 0.001), which compared the control group with the other groups. IAP, intestinal alkaline phosphatase; SC, skim milk culture; SM, skim milk medium.

We hypothesized that the same species of these bacteria could produce SC with high IAP activation capacity. Therefore, we prepared SC samples of *L. delbrueckii* subsp. *jacobsenii* (2 strains), *L. johnsonii* (11 strains), and *S. thermophilus* (22 strains) (Table S2), and measured their IAP activation capacity in Caco-2 cells. The results showed a significant increase in IAP activity in the butyrate, positive control, and almost all SC-treated groups compared with that in the control group. *L. johnsonii* SBT0309 (LJ0309 SC) showed the highest IAP activation capacity (Figure 1).

### 3.2. LJ0309 SC Increases IAP Gene Expression and Protein Levels in Caco-2 Cells

**Figure 2:**
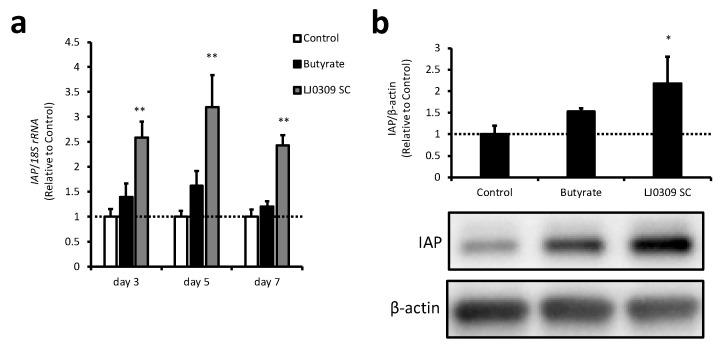
LJ0309 SC increases IAP gene expression and protein levels in Caco-2 cells. IAP (**a**) mRNA and (**b**) protein levels in differentiated Caco-2 cells treated with butyrate (2 mM) or lyophilized LJ0309 SC (5 mg/mL) for 7 days. *IAP* mRNA was analyzed 3 and 5 days after sample addition. The mRNA expression was determined relative to the mean value of the control group at the same time point. IAP and β-actin were detected using immunoblotting and the IAP/β-actin ratio was quantified using densitometry. Data are shown as mean + SD (n = 3) and analyzed using one-way ANOVA followed by Dunnett’s test (* *p* < 0.05, ** *p* < 0.01), which compared the control group with the other groups at the same time point. IAP, intestinal alkaline phosphatase; SC, skim milk culture.

In the screening experiments, we found that LJ0309 SC had a strong IAP activation capacity in Caco-2 cells, so we investigated it further. We found that LJ0309 could produce SC with high IAP-activating capacity using SM without the addition of glucose, sodium ascorbate, and L-cysteine hydrochloride, which are not essential for the growth of LJ0309. Therefore, we used LJ0309 SC prepared without them in the following experiments. The effect of LJ0309 SC on *IAP* gene expression and protein levels in Caco-2 cells was assessed. *IAP* expression significantly increased from day 3 after the addition of LJ0309 SC, and this increase continued until day 7 (Figure 2a). IAP protein levels also significantly increased on day 7 after the addition of LJ0309 SC compared with that in the control group (Figure 2b). These results suggested that LJ0309 SC increased IAP protein levels by upregulating *IAP* gene expression, which contributed to increased IAP activity in Caco-2 cells.

### 3.3. LJ0309 SC Inhibits Inflammatory Response in LPS-Stimulated Caco-2 Cells

**Figure 3:**
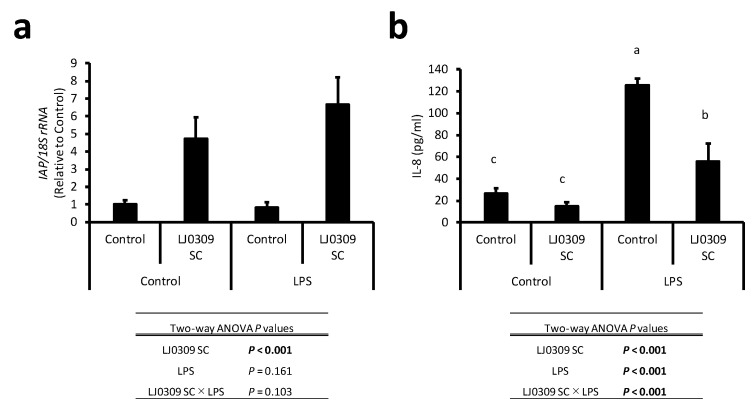
LJ0309 SC inhibited the IL-8 secretion in LPS-stimulated Caco-2 cells. (**a**) *IAP* mRNA and (**b**) IL-8 secretion in differentiated Caco-2 cells treated with LPS (10 µg/mL) and lyophilized LJ0309 SC (5 mg/mL) for 7 days. (**a**) *IAP* mRNA was quantified using qPCR and (**b**) IL-8 levels were determined in the culture supernatant. Data are shown as mean + SD (n = 3) and analyzed using two-way ANOVA followed by Tukey–Kramer’s test (^a, b, c^ *p* < 0.05). LPS, lipopolysaccharide; SC, skim milk culture.

IAP attenuates LPS-induced inflammatory responses [9]. We investigated whether IAP activation by LJ0309 SC reduced the ability of LPS to induce inflammation. LPS and LJ0309 SC were simultaneously added and incubated with Caco-2 cells for three days. The expression of *IAP* in Caco-2 cells and the levels of IL-8 in the medium were measured (Figure 3). We did not detect IL-1β, IL-6, IL-10, and TNF-α using ELISA. In Caco-2 cells, LJ0309 SC increased *IAP* expression both in the presence and absence of LPS (Figure 3a). LPS induced an increase in IL-8 levels in the medium; however, this increase was significantly suppressed by the addition of LJ0309 SC (Figure 3b). Therefore, LJ0309 SC upregulates the *IAP* gene expression of Caco-2 cells in the presence of LPS and suppresses the IL-8 secretion induced by LPS.

### 3.4. LJ0309 SC Increases IAP Gene Expression and Activity in Drosophila Midgut

**Figure 4:**
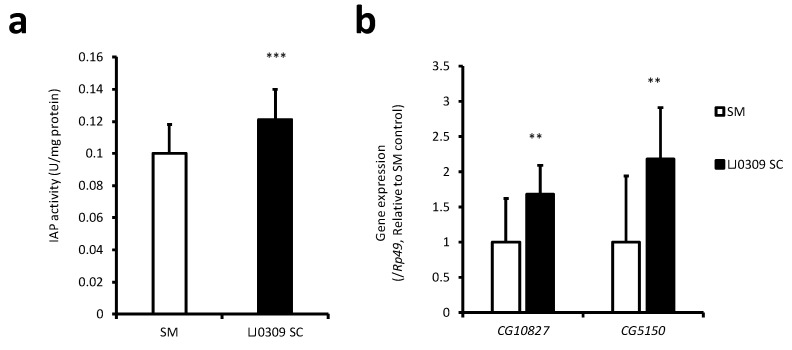
LJ0309 SC increased *IAP* gene expression and activity in the *Drosophila* midgut. (**a**) IAP activity and (**b**) *CG5150* and *CG10827* mRNA expression levels in the midgut of flies fed experimental diets containing 2.5% lyophilized SM or 2.5% lyophilized LJ0309 SC for 5 weeks. Data are shown as mean + SD [(**a**) n = 24, 25 (72, 75 flies), (**b**) n = 12, 13 (60, 65 flies)] and analyzed using Student’s *t*-test (** *p* < 0.01, *** *p* < 0.001). SC, skim milk culture; SM, skim milk medium.

Although we showed that LJ0309 SC directly activated IAP in intestinal epithelial cells, its effect on IAP in the gut is unknown. IAP activity in Caco-2 cells and the *Drosophila* midgut shows similar responses to dietary components [24]. Thus, the effect of the LJ0309 SC on IAP activity and gene expression in the *Drosophila* midgut was investigated. *Drosophila* were reared for 5 weeks on a diet containing 2.5% lyophilized SM or lyophilized LJ0309 SC, and IAP activity and gene expression were measured in the midgut. The results showed a significant increase in IAP activity in the LJ0309 SC-fed group compared with the SM-fed group (Figure 4a). The expression levels of *CG5150* and *CG10827*, fly homologs of human *IAP* genes, were also significantly higher in the LJ0309 SC-fed group than in the SM-fed group (Figure 4b). Therefore, LJ0309 SC increased the expression of IAP homologues and enhanced IAP activity in the *Drosophila* midgut.

## 4. Discussion

In a previous study, fermented milk produced from *L. delbrueckii* subsp. *bulgaricus* and *S. thermophilus* was reported to have an IAP-activating capacity in vivo [21]. In this study, we investigated whether IAP could be activated in cultures of other bacteria and carried out SC screening using various lactic acid bacteria and bifidobacteria. The results showed a relatively strong effect on the SC samples prepared with *L. delbrueckii* subsp. *jakobsenii*, *S. thermophilus,* and *L. johnsonii*. In the present in vitro screening using Caco-2 cells, IAP-activating effects were observed in the same bacterial species, *L. delbrueckii* (although the subspecies differ) and *S. thermophilus*, as in a previous in vivo report [21]. To the best of our knowledge, this study is the first time the IAP-activating capacity of *L. johnsonii* SC was reported.

In the screening experiment, we found the highest IAP-activating capacity in LJ0309 SC; however, the active ingredient is not yet known. Lactic acid is one of the most common metabolites produced during fermentation by many lactic acid bacteria, including LJ0309. Lactic acid affects intestinal epithelial cell proliferation and differentiation of intestinal epithelial cells [25]. IAP is an enzyme whose expression increases during intestinal epithelial cell differentiation [26]; therefore, it was hypothesized that the lactic acid produced by lactic acid bacteria could affect the differentiation of intestinal epithelial cells and increase their IAP activity. However, this study used differentiated Caco-2 cells, which showed a plateau in IAP due to differentiation. Thus, in the present study, the effect of increased IAP activity on lactate-induced differentiation was small.

It is possible that lactate contributes directly to IAP activation rather than to differentiation. Therefore, we tested whether lactic acid affects IAP activity in differentiated Caco-2 cells. Lactic acid contains D- and L-optical isomers; therefore, D-lactate and L-lactic acid were evaluated. The results showed that L-lactate had no IAP-activating capacity, whereas D-lactate did (Figure S3), suggesting that D-lactic acid may contribute to the IAP-activating capacity of SC.

D-lactic acid is produced by a relatively large number of lactic acid bacteria species [27] and is not characteristic of any particular lactic acid bacteria. For example, *L. johnsonii* produces D- and L-lactic acid heterogeneously [28]. In contrast, *L. delbrueckii* only produces D-lactic acid [27]. The D-lactic acid concentration of the SC used in this study was measured to be 87.1 mM for LJ0309 SC and 161.5 mM for SC produced with *L. delbrueckii* subsp. *jakobsenii* SBT0803, a selected strain in the screening experiment, as well as LJ0309. This indicated that LJ0309 did not produce the highest amount of D-lactate in the screening experiment. Furthermore, the final concentration of D-lactate in the medium (approximately 4 mM) when LJ0309 SC was added to Caco-2 cells was the concentration at which IAP was not activated (Figure S3). Although D-lactate may contribute to the IAP-activating potential of LJ0309 SC, it has been suggested that there are other IAP-activating factors besides D-lactate.

Bacterial cells are the characteristic components of various SCs. There have been many reports of bacterial cells acting as active ingredients in Caco-2 cells. Peng et al. screened bacterial cells that inhibited *Staphylococcus aureus* translocation in a Caco-2 monolayer model and found particularly strong effects in *Limosilactobacillus fermentum* NCU3087 and NCU3088 [29]. Liu et al. also reported that bacterial cells strengthened the barrier function of Caco-2 cells and found that the effect varied among strains, with some strains strengthening the barrier and others weakening it [30]. The varied effects of bacterial cells on the physiological functions of intestinal epithelial cells suggests that bacterial cells may be a characteristic active ingredient of LJ0309 SC. Therefore, we prepared the LJ0309 cell powder with the defined medium and evaluated the IAP-activating capacity of the bacterial cells in Caco-2 cells, and it was found that 0.5 g/mL LJ0309 cells can increase IAP activity as well as 5 mg/mL LJ0309 SC (Figure S4). The live cell content was 3.8 × 10^9^ CFU/g powder in lyophilized LJ0309 SC powder and 1.0 × 10^11^ CFU/g powder in lyophilized LJ0309 cell powder, suggesting that LJ0309 SC powder may contain approximately 4% (*w*/*w*) LJ0309 cells. Although these results do not fully explain the effects of LJ0309 SC, they suggest that LJ0309 cells contribute to the IAP activation capacity of LJ0309 SC.

Peptides and amino acids may also be involved in the IAP-activating capacity of the LJ0309 SC. For example, the IIAEK peptide, lactostatin, activates IAP [31]. Additionally, tryptophan and phenylalanine inhibit IAP [20,32]. Further analyses, such as amino acid and peptide analyses in SC, may reveal a relationship between amino acid/peptide ratios and IAP activity.

In the present study, we showed that LJ0309 SC increased IAP protein levels by upregulating *IAP* gene expression, suggesting that it contributes to IAP activation. The transcription factors that bind to the IAP promoter include HNF-4α [33] KLF4 [34], KLF5 [35], thyroid hormone receptor [36], ZBP-89 [37], and Cdx-1/2 [38]. The compounds in the LJ0309 SC may act on these transcription factors and increase IAP expression. For example, lactate affects the transcriptional activity of KLF5 [39]. As mentioned above, lactate is one of the components that may be involved in the activity of LJ0309 SC and KLF5 activation by lactate may contribute to increased IAP expression in LJ0309 SC.

In this study, LJ0309 SC activated IAP and inhibited LPS-induced inflammation. IAP removes phosphate groups from LPS, an inflammatory substance derived from Gram-negative bacteria [7]. Lipid A, the active body of LPS, has a reduced ability to induce inflammation via the TLR4/MD2 pathway when its phosphate groups are released [40]. In fact, the inflammatory capacity of LPS treated with IAP was reduced [9]. Therefore, the inhibitory effect of the LJ0309 SC on LPS-induced inflammation observed in the present study may be mediated by IAP activation.

Toll-like receptor signaling might be involved in the mechanism through which LJ0309SC inhibited LPS-induced inflammation. Lipopolysaccharide acts as a TLR4 agonist that activates inflammatory pathways such as nuclear factor kappa B (NFκΒ) and induces pro-inflammatory gene expression [6]. Other TLR agonists such as the TLR2 agonist lipoteichoic acid (LTA) inhibit the LPS activation of inflammatory pathways [41,42]. *Lactobacillus* spp. harbors the TLR2 agonists peptidoglycan and LTA [41,42]. Thus, *Lactobacillus johnsonii* SBT0309 might inhibit LPS activation of the inflammatory pathway via TLR2 signaling.

We evaluated the anti-inflammatory effects of LJ0309 SC on LPS with a focus on IAP, although some SCs have been reported to inhibit LPS-induced inflammation via pathways other than IAP activation. For example, intraperitoneal administration of LPS increases the serum levels of inflammatory cytokines; however, this increase is suppressed in rats fed a milk culture of *Limosilactobacillus fermentum* [43]. Serum levels of the anti-inflammatory cytokine IL-10 were increased in these rats [43]. The addition of *Lacticaseibacillus rhamnosus* R0011 milk culture to macrophage cell lines also inhibits the increase in LPS-stimulated pro-inflammatory M1-type macrophages and promotes an increase in anti-inflammatory M2-type macrophages that produce IL-10 [44]. These studies focused on IL-10, rather than IAP, as a mechanism underlying the anti-inflammatory effects of milk cultures. Caco-2 cells express *IL-10* mRNA [45,46]. We considered that IL-10 might be involved in the anti-inflammatory effect of LJ0309 SC and measured *IL-10* gene and protein levels, which were not detected. Under our experimental conditions, the contribution of IL-10 to the inhibitory effect of LJ0309 SC on LPS-induced inflammation was expected to be small.

We demonstrated that LJ0309 SC can directly increase IAP activity in a Caco-2 cell culture model, but it was not known whether LJ0309 SC had a similar effect in the gut when consumed as a meal. We used *D. melanogaster*, an alternative organism to animal models of the gut, to test whether LJ0309 SC could increase IAP activity in the gut. LJ0309 SC increased IAP activity and gene expression in the *Drosophila* midgut, as well as in Caco-2 cells. Although little is known about IAP activity in the *Drosophila* midgut, it has been suggested to act as a gut mucosal defense factor to prevent intestinal permeability in *Drosophila* [24]. Kühn et al. found that IAP activity in *Drosophila* midgut decreases with age and double knockdown of IAP homologues (*CG5150* and *CG10827*) shortens the lifespan [12], suggesting that IAP may affect the lifespan of *Drosophila*. This study did not test the effect of LJ0309 SC consumption on intestinal permeability or lifespan in *Drosophila*; however, future studies should evaluate its effects on these parameters.

In the present study, we demonstrated that LJ0309 SC has a potent IAP-activating capacity in a screening experiment using Caco-2 cells. This effect is thought to be due to increased *IAP* gene expression and protein levels. We also demonstrated that LJ0309 upregulated *IAP* gene expression and inhibited pro-inflammatory cytokine responses in LPS-stimulated Caco-2 cells. Moreover, IAP activation by the LJ0309 SC was demonstrated in *D. melanogaster*. These results were obtained only in cell culture and *Drosophila* models and need to be validated in mammalian animal models. Although further in vivo studies are required, LJ0309 SC might help to ameliorate LPS-induced inflammation and disease via IAP activation.

## Data Availability

The original contributions presented in this study are included in the article/Supplementary Materials. Further inquiries can be directed to the corresponding author.

## Supplementary Materials

The following supporting information can be downloaded at https://www.mdpi.com/article/10.3390/cells14050358/s1. Figure S1: Screening of SC samples produced with 128 strains of 55 bacterial species to increase the IAP activity of Caco-2 cells; Figure S2: Screening of the selected SC samples to increase IAP activity of Caco-2 cells; Figure S3: D-lactate increased IAP activity in Caco-2 cells; Figure S4: LJ0309 cells increased IAP activity in Caco-2 cells; Table S1: List of bacteria used for SC samples; Table S2: List of Streptococcus thermophilus, Lactobacillus delbrueckii subsp. jakobsenii, and Lactobacillus johnsonii used for SC samples; Table S3: List of qPCR primers.
