# Supplementary material for: Skim Milk Culture of Lactobacillus johnsonii SBT0309 Increases Intestinal Alkaline Phosphatase Activity and Inhibits Lipopolysaccharide-Induced Interleukin-8 Production in Intestinal Epithelial Cells
Source: Cells. 2025 Feb 28;14(5):358. doi: 10.3390/cells14050358 (PMC11898809; doi:10.3390/cells14050358)
Supplement: Supplementary file 1 [file cells-14-00358-s001.zip › cells-3451941-supplementary.pdf]

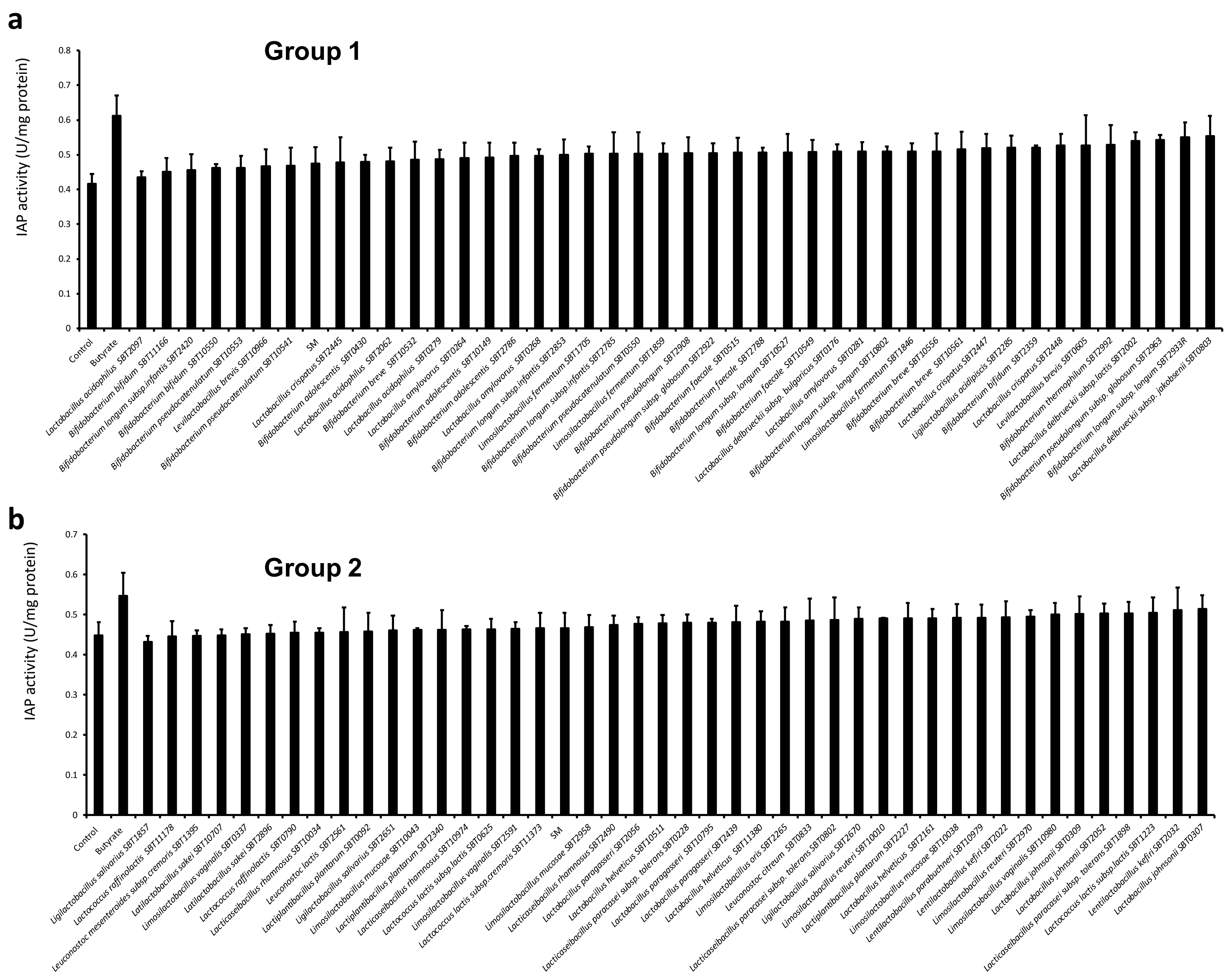
a
Group 1
IAP activity (U/mg protein)
0
0.1
0.2
0.3
0.4
0.5
0.6
0.7
0.8
Control
Butyrate
Lactobacillus acidophilus SBT2097
Bifidobacterium bifidum SBT11166
Bifidobacterium longum subsp.infantis SBT2420
Bifidobacterium bifidum SBT10550
Bifidobacterium pseudocatenulatum SBT10553
Levilactobacillus brevis SBT10966
Bifidobacterium pseudocatenulatum SBT10541
SM
Lactobacillus crispatus SBT2445
Bifidobacterium adolescentis SBT0430
Lactobacillus acidophilus SBT2062
Bifidobacterium breve SBT10532
Lactobacillus acidophilus SBT0279
Lactobacillus amylovorus SBT0264
Bifidobacterium adolescentis SBT10149
Bifidobacterium adolescentis SBT2786
Lactobacillus amylovorus SBT0268
Bifidobacterium longum subsp.infantis SBT2853
Limosilactobacillus fermentum SBT1705
Bifidobacterium longum subsp.infantis SBT2785
Bifidobacterium pseudocatenulatum SBT0550
Limosilactobacillus fermentum SBT1859
Bifidobacterium pseudolongum SBT2908
Bifidobacterium pseudolongum subsp. globosum SBT2922
Bifidobacterium faecale SBT0515
Bifidobacterium faecale SBT2788
Bifidobacterium longum subsp. longum SBT10527
Bifidobacterium faecale SBT10549
Lactobacillus delbrueckii subsp. bulgaricus SBT0176
Lactobacillus amylovorus SBT0281
Bifidobacterium longum subsp. longum SBT10802
Limosilactobacillus fermentum SBT1846
Bifidobacterium breve SBT10556
Bifidobacterium breve SBT10561
Lactobacillus crispatus SBT2447
Ligilactobacillus acidipiscis SBT2285
Bifidobacterium bifidum SBT2359
Lactobacillus crispatus SBT2448
Levilactobacillus brevis SBT0605
Bifidobacterium thermophilum SBT2992
Lactobacillus delbrueckii subsp.lactis SBT2002
Bifidobacterium pseudolongum subsp. globosum SBT2963
Bifidobacterium longum subsp. longum SBT2933R
Lactobacillus delbrueckii subsp. jakobsenii SBT0803
b
Group 2
IAP activity (U/mg protein)
0
0.1
0.2
0.3
0.4
0.5
0.6
0.7
Control
Butyrate
Ligilactobacillus salivarius SBT1857
Lactococcus raffinolactis SBT11178
Leuconostoc mesenteroides subsp.cremoris SBT1395
Latilactobacillus sakei SBT10707
Limosilactobacillus vaginalis SBT0337
Latilactobacillus sakei SBT2896
Lactococcus raffinolactis SBT0790
Lacticaseibacillus rhamnosus SBT10034
Leuconostoc lactis SBT2561
Lactiplantibacillus plantarum SBT0092
Ligilactobacillus salivarius SBT2651
Limosilactobacillus mucosae SBT10043
Lactiplantibacillus plantarum SBT2340
Lacticaseibacillus rhamnosus SBT10974
Lactococcus lactis subsp.lactis SBT0625
Limosilactobacillus vaginalis SBT2591
Lactococcus lactis subsp.cremoris SBT11373
SM
Limosilactobacillus mucosae SBT2958
Lacticaseibacillus rhamnosus SBT2490
Lactobacillus paragasseri SBT2056
Lactobacillus helveticus SBT10511
Lacticaseibacillus paracasei subsp. tolerans SBT0228
Lactobacillus paragasseri SBT10795
Lactobacillus paragasseri SBT2439
Lactobacillus helveticus SBT11380
Limosilactobacillus oris SBT2265
Leuconostoc citreum SBT0833
Lacticaseibacillus paracasei subsp. tolerans SBT0802
Ligilactobacillus salivarius SBT2670
Limosilactobacillus reuteri SBT10010
Lactiplantibacillus plantarum SBT2227
Lactobacillus helveticus SBT2161
Limosilactobacillus mucosae SBT10038
Lentilactobacillus parabuchneri SBT10979
Lentilactobacillus kefiri SBT2022
Limosilactobacillus reuteri SBT2970
Limosilactobacillus vaginalis SBT10980
Lactobacillus johnsonii SBT0309
Lactobacillus johnsonii SBT2052
Lacticaseibacillus paracasei subsp. tolerans SBT1898
Lactococcus lactis subsp.lactis SBT1223
Lentilactobacillus kefiri SBT2032
Lactobacillus johnsonii SBT0307

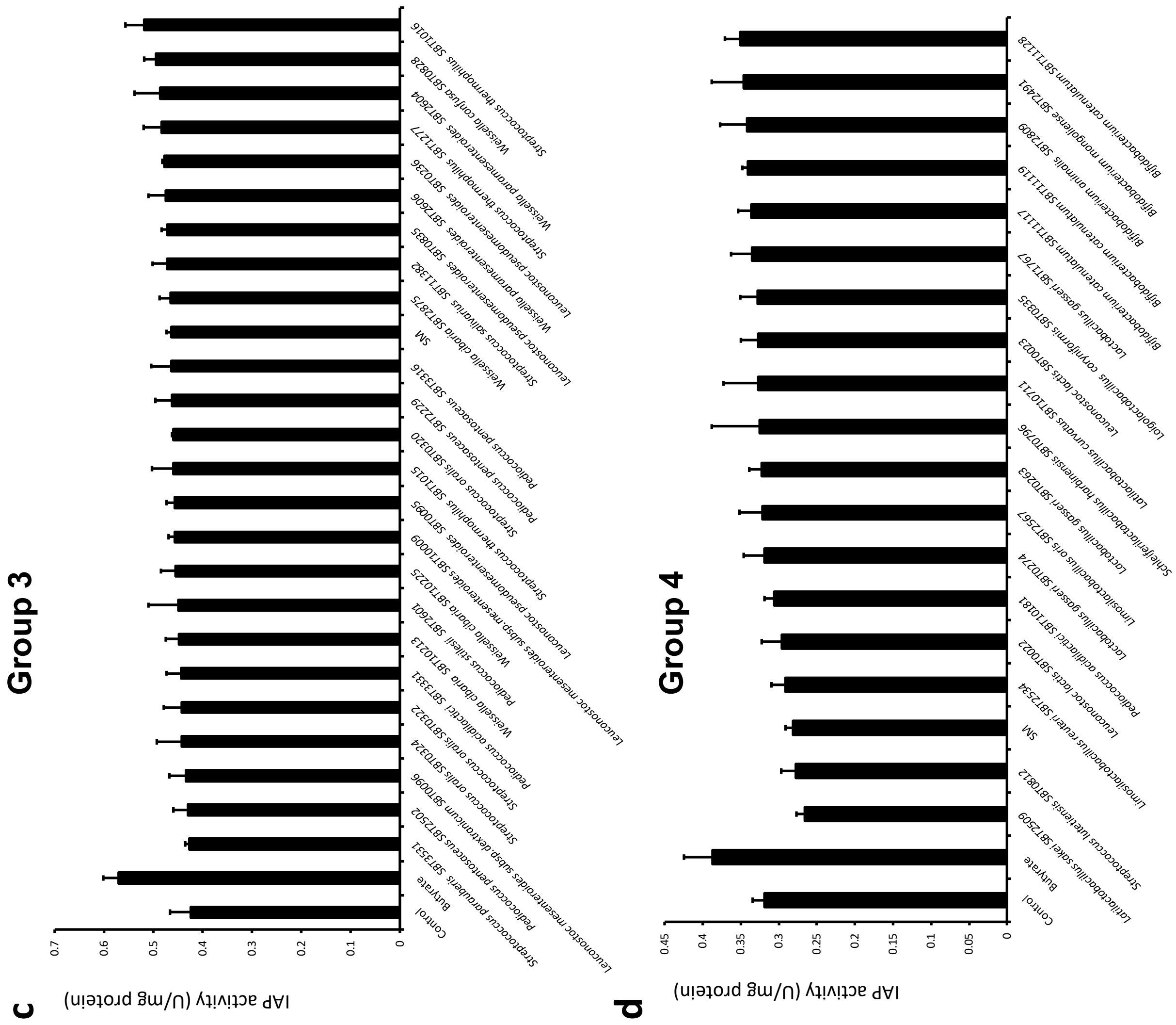

Figure S1. Screening of SC samples produced with 128 strains of 55 bacterial species to increase IAP activity of Caco-2 cells. IAP activity in differentiated Caco-2 cells treated with butyrate (2 mM), lyophilized SM (1 mg/mL) or lyophilized SCs (1 mg/mL) for 7 days. The SC samples were divided into four groups (**(a)** Group 1: 43 strains; **(b)** Group 2: 43 strains; **(c)** Group 3: 24 strains; **(d)** Group 4: 18 strains). SC samples were prepared with lactic acid bacteria and bifidobacteria as listed in Table S1. Data are shown as mean + SD (n = 3).

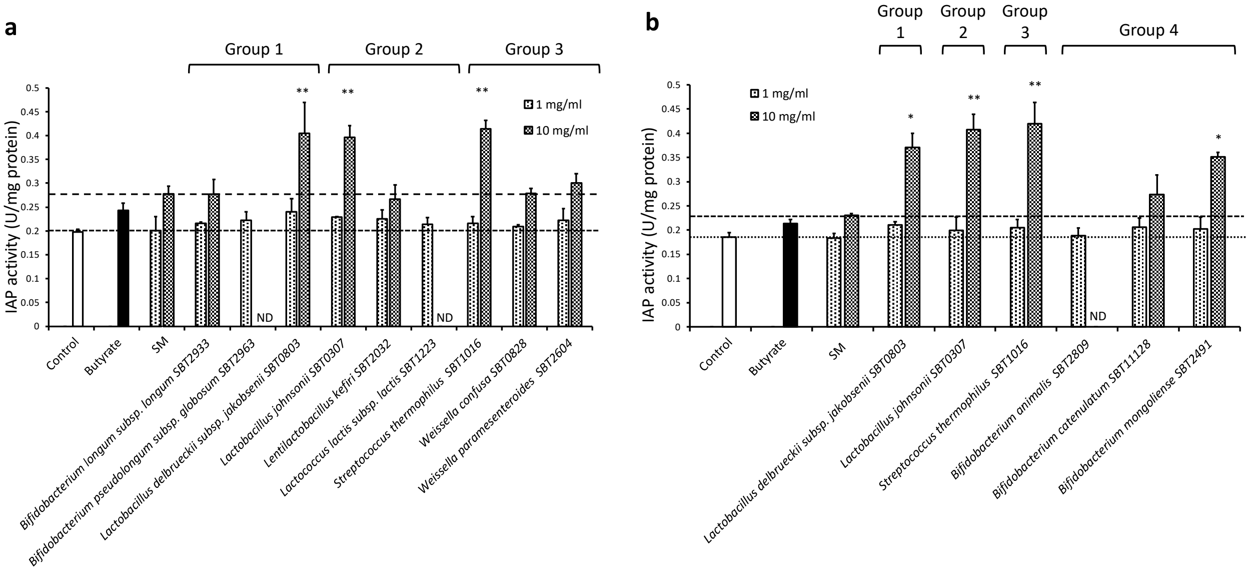

Figure S2. Screening of the selected SC samples to increase IAP activity of Caco-2 cells. IAP activity in differentiated Caco-2 cells treated with butyrate (2 mM), lyophilized SM (1, 10 mg/mL) or lyophilized SCs (1, 10 mg/mL) for 7 days. Three SC samples with the highest IAP activation capacity were selected from each group defined in Figure S1 (Groups 1-4). **(a)** The SC samples selected from Group 1, Group 2 and Group 3 were compared. **(b)** The SC samples selected from Group 4 were compared with those selected from Groups 1-3. ND means that IAP activity could not be detected because the cells were detached during the incubation period. Data are shown as mean + SD (n = 3) and analyzed by one-way ANOVA followed by Dunnett's test ($^{*}P < 0.05$, $^{**}P < 0.01$), which compared the SM-treated group with the other groups at the same dose.

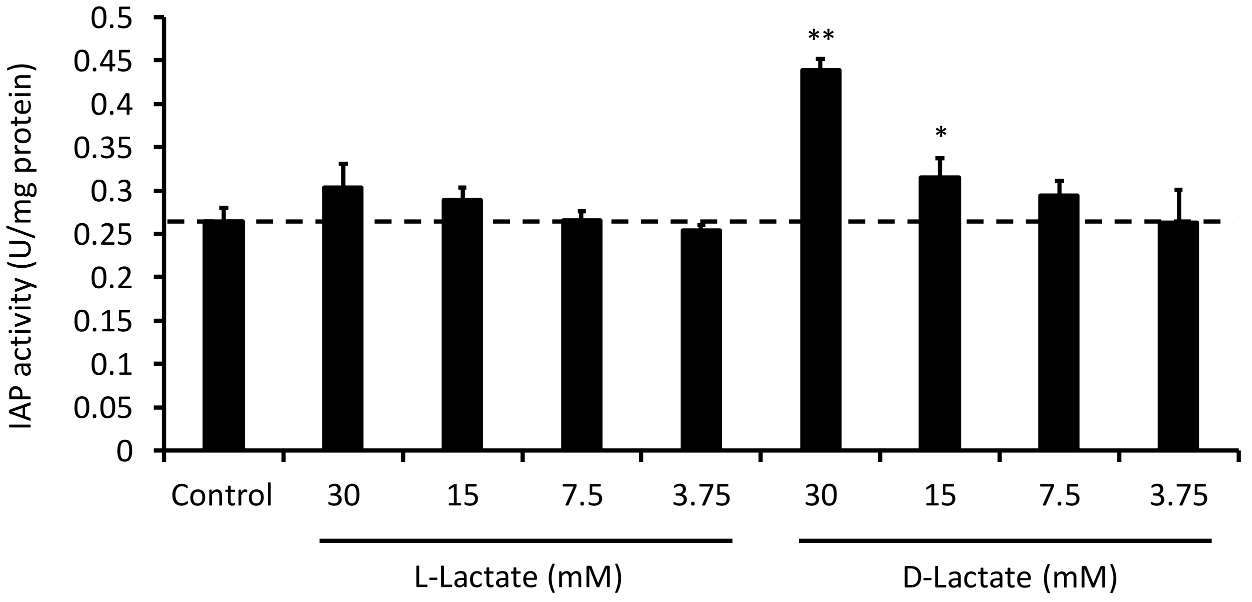

Figure S3. D-lactate increased IAP activity in Caco-2 cells. IAP activity in differentiated Caco-2 cells treated with butyrate (2 mM), L-lactate (3.75-30 mM) or D-lactate (3.75-30 mM) for 7 days. Data are shown as mean + SD (n = 3) and analyzed by one-way ANOVA followed by Dunnett's test ($^{*}P < 0.05$, $^{**}P < 0.01$), which compared the control group with the other groups.

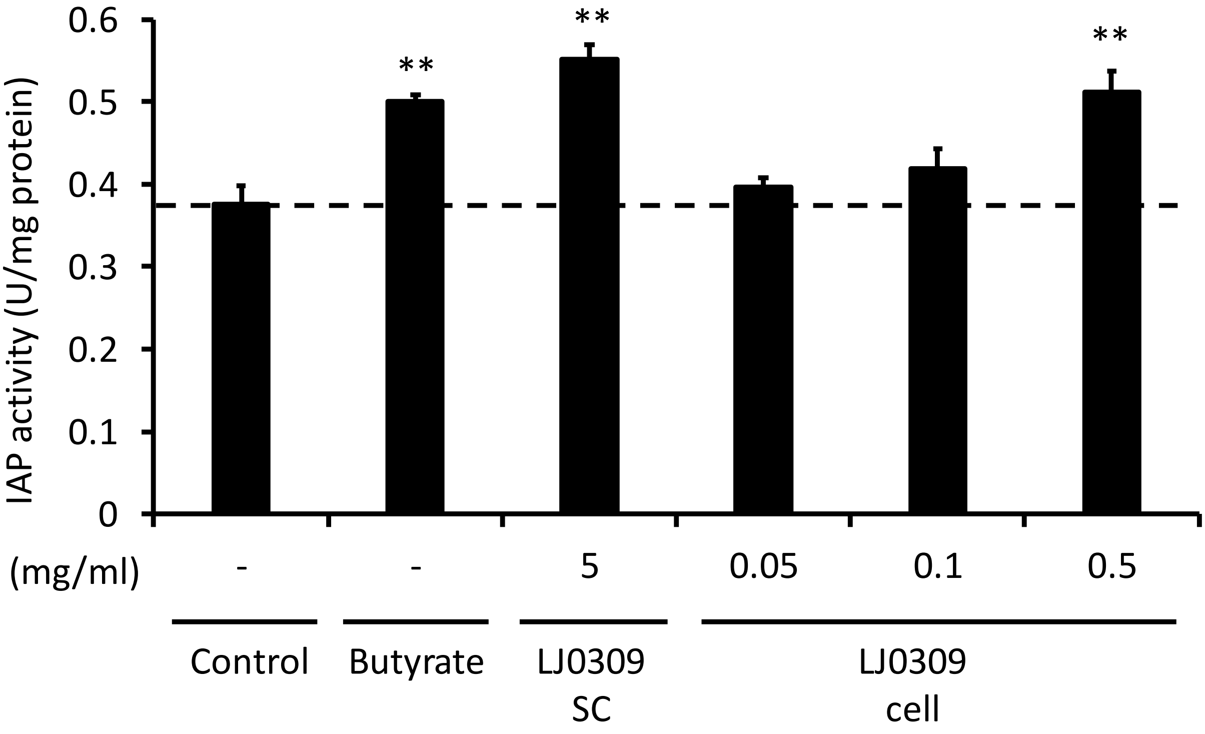

Figure S4. LJ0309 cells increased IAP activity in Caco-2 cells. IAP activity in differentiated Caco-2 cells treated with butyrate (2 mM), lyophilized LJ0309 SC (5 mg/mL) or lyophilized LJ0309 cells (0.05-0.5 mg/mL) for 7 days. LJ0309 cells were obtained as follows: cultured with De Man, Rogosa, and Sharpe (MRS) broth, collected by centrifugation, washed twice with saline, once with sterile water and lyophilized. The live cell content was 3.8×10^{9} cfu/g powder in lyophilized LJ0309 SC powder and 1.0×10^{11} cfu/g powder in lyophilized LJ0309 cell powder. Data are shown as mean + SD (n = 3) and analyzed by one-way ANOVA followed by Dunnett's test ($^{**}P < 0.01$), which compared the control group with the other groups.

Table S1 List of bacteria used for SC samples

Strain name	Defined medium	pH value of SC	Group No.
Bifidobacterium adolescentis SBT0430	GAM+G	4.35	Group 1
Bifidobacterium adolescentis SBT2786	GAM+G	4.22	Group 1
Bifidobacterium adolescentis SBT10149	GAM+G	4.22	Group 1
Bifidobacterium bifidum SBT2359	GAM+G	4.29	Group 1
Bifidobacterium bifidum SBT10550	GAM+G	4.7	Group 1
Bifidobacterium bifidum SBT11166	GAM+G	4.17	Group 1
Bifidobacterium breve SBT10532	GAM+G	4.3	Group 1
Bifidobacterium breve SBT10561	GAM+G	3.99	Group 1
Bifidobacterium breve SBT10556	GAM+G	4.36	Group 1
Bifidobacterium faecale SBT0515	GAM+G	4.21	Group 1
Bifidobacterium faecale SBT2788	GAM+G	4.19	Group 1
Bifidobacterium faecale SBT10549	GAM+G	4.05	Group 1
Bifidobacterium longum subsp. *longum* SBT2933R	GAM+G	3.94	Group 1
Bifidobacterium longum subsp. *longum* SBT10527	GAM+G	4.08	Group 1
Bifidobacterium longum subsp. *longum* SBT10802	GAM+G	4.24	Group 1
Bifidobacterium longum subsp. *infantis* SBT2420	GAM+G	3.88	Group 1
Bifidobacterium longum subsp. *infantis* SBT2785	GAM+G	4.39	Group 1
Bifidobacterium longum subsp. *infantis* SBT2853	GAM+G	4.54	Group 1
Bifidobacterium pseudocatenulatum SBT0550	GAM+G	4.04	Group 1
Bifidobacterium pseudocatenulatum SBT10541	GAM+G	4.08	Group 1

Bifidobacterium pseudocatenulatum SBT10553	GAM+G	4.15	Group 1
Bifidobacterium pseudolongum SBT2908	GAM+G	4.13	Group 1
Bifidobacterium pseudolongum subsp. *globosum* SBT2922	GAM+G	4.19	Group 1
Bifidobacterium pseudolongum subsp. *globosum* SBT2963	GAM+G	4.27	Group 1
Bifidobacterium thermophilum SBT2992	GAM+G	4.11	Group 1
Ligilactobacillus acidipiscis SBT2285	MRS	5.84	Group 1
Lactobacillus acidophilus SBT0279	MRS	3.71	Group 1
Lactobacillus acidophilus SBT2062	MRS	3.62	Group 1
Lactobacillus acidophilus SBT2097	MRS	3.72	Group 1
Lactobacillus amylovorus SBT0264	MRS	3.94	Group 1
Lactobacillus amylovorus SBT0268	MRS	3.77	Group 1
Lactobacillus amylovorus SBT0281	MRS	4.27	Group 1
Levilactobacillus brevis SBT0605	MRS	4.86	Group 1
Levilactobacillus brevis SBT10966	MRS	5.73	Group 1
Lactobacillus crispatus SBT2445	MRS	4.11	Group 1
Lactobacillus crispatus SBT2447	MRS	4.43	Group 1
Lactobacillus crispatus SBT2448	MRS	4.24	Group 1
Lactobacillus delbrueckii subsp. *bulgaricus* SBT0176	MRS	3.31	Group 1
Lactobacillus delbrueckii subsp. *jakobsenii* SBT0803	MRS	3.39	Group 1
Lactobacillus delbrueckii subsp. *lactis* SBT2002	MRS	3.43	Group 1
Limosilactobacillus fermentum SBT1705	MRS	5.02	Group 1
Limosilactobacillus fermentum SBT1846	MRS	4.91	Group 1
Limosilactobacillus fermentum SBT1859	MRS	4.96	Group 1

Lactobacillus paragasseri SBT2056	MRS	3.79	Group 2
Lactobacillus paragasseri SBT2439	MRS	3.84	Group 2
Lactobacillus paragasseri SBT10795	MRS	3.77	Group 2
Lactobacillus helveticus SBT2161	MRS	3.25	Group 2
Lactobacillus helveticus SBT11380	MRS	3.58	Group 2
Lactobacillus helveticus SBT10511	MRS	3.43	Group 2
Lactobacillus johnsonii SBT0307	MRS	3.37	Group 2
Lactobacillus johnsonii SBT0309	MRS	3.43	Group 2
Lactobacillus johnsonii SBT2052	MRS	3.69	Group 2
Lentilactobacillus kefiri SBT2022	MRS	5.04	Group 2
Lentilactobacillus kefiri SBT2032	MRS	5.26	Group 2
Limosilactobacillus mucosae SBT2958	MRS	5.05	Group 2
Limosilactobacillus mucosae SBT10038	MRS	4.65	Group 2
Limosilactobacillus mucosae SBT10043	MRS	4.82	Group 2
Limosilactobacillus oris SBT2265	MRS	5.13	Group 2
Lentilactobacillus parabuchneri SBT10979	MRS	4.94	Group 2
Lacticaseibacillus paracasei subsp. *tolerans* SBT0228	MRS	4.02	Group 2
Lacticaseibacillus paracasei subsp. *tolerans* SBT0802	MRS	4.3	Group 2
Lacticaseibacillus paracasei subsp. *tolerans* SBT1898	MRS	4.18	Group 2
Lactiplantibacillus plantarum SBT0092	MRS	4.54	Group 2
Lactiplantibacillus plantarum SBT2227	MRS	4.7	Group 2
Lactiplantibacillus plantarum SBT2340	MRS	4.77	Group 2
Limosilactobacillus reuteri SBT2970	MRS	4.87	Group 2

Limosilactobacillus reuteri SBT10010	MRS	5.07	Group 2
Lacticaseibacillus rhamnosus SBT2490	MRS	3.87	Group 2
Lacticaseibacillus rhamnosus SBT10034	MRS	3.99	Group 2
Lacticaseibacillus rhamnosus SBT10974	MRS	4.07	Group 2
Latilactobacillus sakei SBT2896	MRS	5.06	Group 2
Latilactobacillus sakei SBT10707	MRS	4.78	Group 2
Ligilactobacillus salivarius SBT2651	MRS	3.98	Group 2
Ligilactobacillus salivarius SBT1857	MRS	4.36	Group 2
Ligilactobacillus salivarius SBT2670	MRS	4.12	Group 2
Limosilactobacillus vaginalis SBT0337	MRS	5.18	Group 2
Limosilactobacillus vaginalis SBT2591	MRS	5.41	Group 2
Limosilactobacillus vaginalis SBT10980	MRS	5.44	Group 2
Lactococcus lactis subsp. *lactis* SBT0625	M17	4.18	Group 2
Lactococcus lactis subsp. *lactis* SBT1223	M17	4.15	Group 2
Lactococcus lactis subsp. *cremoris* SBT11373	M17	4.25	Group 2
Lactococcus raffinolactis SBT0790	M17	4.45	Group 2
Lactococcus raffinolactis SBT11178	M17	4.19	Group 2
Leuconostoc citreum SBT0833	MRS	4.91	Group 2
Leuconostoc lactis SBT2561	MRS	5.03	Group 2
Leuconostoc mesenteroides subsp. *cremoris* SBT1395	MRS	4.8	Group 2
Leuconostoc mesenteroides subsp. *dextranicum* SBT0096	MRS	4.91	Group 3
Leuconostoc mesenteroides subsp. *mesenteroides* SBT10009	MRS	4.79	Group 3
Leuconostoc pseudomesenteroides SBT0095	MRS	5.09	Group 3

Leuconostoc pseudomesenteroides SBT0236	MRS	5.06	Group 3
Leuconostoc pseudomesenteroides SBT0835	MRS	4.96	Group 3
Pediococcus acidilactici SBT3331	MRS	5.13	Group 3
Pediococcus pentosaceus SBT2229	MRS	4.97	Group 3
Pediococcus pentosaceus SBT2502	MRS	4.68	Group 3
Pediococcus pentosaceus SBT3316	MRS	5.14	Group 3
Pediococcus stilesii SBT2601	MRS	5.06	Group 3
Streptococcus oralis SBT0320	MRS	4.06	Group 3
Streptococcus oralis SBT0322	MRS	4.09	Group 3
Streptococcus oralis SBT0324	MRS	4.08	Group 3
Streptococcus parauberis SBT3531	GAM+G	4.71	Group 3
Streptococcus salivarius SBT11382	MRS	4.35	Group 3
Streptococcus thermophilus SBT1015	M17+G	4.17	Group 3
Streptococcus thermophilus SBT1016	M17+G	4.16	Group 3
Streptococcus thermophilus SBT1277	M17+G	4.36	Group 3
Weissella cibaria SBT2875	MRS	4.79	Group 3
Weissella cibaria SBT10213	MRS	4.93	Group 3
Weissella cibaria SBT10225	MRS	4.77	Group 3
Weissella confusa SBT0828	MRS	4.91	Group 3
Weissella paramesenteroides SBT2604	MRS	4.95	Group 3
Weissella paramesenteroides SBT2606	MRS	5.03	Group 3
Bifidobacterium animalis SBT2809	GAM+G	4.23	Group 4
Bifidobacterium catenulatum SBT11117	GAM+G	4.27	Group 4

Bifidobacterium catenulatum SBT11119	GAM+G	4.09	Group 4
Bifidobacterium catenulatum SBT11128	GAM+G	4.08	Group 4
Bifidobacterium mongoliense SBT2491	GAM+G	4.17	Group 4
Loigolactobacillus coryniformis SBT0335	MRS	4.87	Group 4
Latilactobacillus curvatus SBT10711	MRS	4.96	Group 4
Lactobacillus gasseri SBT0263	MRS	4	Group 4
Lactobacillus gasseri SBT0274	MRS	4.1	Group 4
Lactobacillus gasseri SBT1767	MRS	4.3	Group 4
Schleiferilactobacillus harbinensis SBT0796	MRS	4.79	Group 4
Limosilactobacillus oris SBT2567	MRS	5.05	Group 4
Limosilactobacillus reuteri SBT2534	MRS	5.14	Group 4
Latilactobacillus sakei SBT2509	MRS	5.13	Group 4
Leuconostoc lactis SBT0022	MRS	5.18	Group 4
Leuconostoc lactis SBT0023	MRS	5.12	Group 4
Pediococcus acidilactici SBT10181	MRS	4.77	Group 4
Streptococcus lutetiensis SBT0812	MRS	4.08	Group 4

MRS: De Man, Rogosa, and Sharpe broth, GAM: Gifu Anaerobic Medium

M17+G: M17 broth with 1% glucose, GAM+G: GAM broth with 1% glucose

Table S2. List of *Streptococcus thermophilus, Lactobacillus delbrueckii* subsp. *jakobsenii* and *Lactobacillus johnsonii* used for SC samples

Strain name	Defined medium	pH value of SC
Streptococcus thermophilus SBT0055	M17+G	4.28
Streptococcus thermophilus SBT0084	M17+G	4.42
Streptococcus thermophilus SBT0087	M17+G	4.21
Streptococcus thermophilus SBT0101	M17+G	4.06
Streptococcus thermophilus SBT0109	M17+G	4.33
Streptococcus thermophilus SBT0111	M17+G	4.03
Streptococcus thermophilus SBT0148	M17+G	4.15
Streptococcus thermophilus SBT0156	M17+G	4.07
Streptococcus thermophilus SBT0187	M17+G	4.11
Streptococcus thermophilus SBT1007	M17+G	4.15
Streptococcus thermophilus SBT1021A	M17+G	4.16
Streptococcus thermophilus SBT1054	M17+G	4.6
Streptococcus thermophilus SBT1055	M17+G	4.28
Streptococcus thermophilus SBT1095	M17+G	4.12
Streptococcus thermophilus SBT1190	M17+G	4.37
Streptococcus thermophilus SBT1348	M17+G	4.34
Streptococcus thermophilus SBT1380	M17+G	4.4
Streptococcus thermophilus SBT10134	M17+G	4.08
Streptococcus thermophilus SBT10137	M17+G	4.09
Streptococcus thermophilus SBT1015	M17+G	4.17

Streptococcus thermophilus SBT1016	M17+G	4.16
Streptococcus thermophilus SBT1277	M17+G	4.36
Lactobacillus delbrueckii subsp. *jakobsenii* SBT0813	MRS	3.66
Lactobacillus delbrueckii subsp. *jakobsenii* SBT0803	MRS	3.39
Lactobacillus johnsonii SBT0075	MRS	3.61
Lactobacillus johnsonii SBT0277	MRS	3.96
Lactobacillus johnsonii SBT0305	MRS	3.58
Lactobacillus johnsonii SBT2058	MRS	3.94
Lactobacillus johnsonii SBT2075	MRS	3.75
Lactobacillus johnsonii SBT2078	MRS	3.65
Lactobacillus johnsonii SBT2079	MRS	6.61
Lactobacillus johnsonii SBT2082	MRS	3.59
Lactobacillus johnsonii SBT0307	MRS	3.37
Lactobacillus johnsonii SBT0309	MRS	3.43
Lactobacillus johnsonii SBT2052	MRS	3.69

MRS: De Man, Rogosa, and Sharpe broth, M17+G: M17 broth with 1% glucose

Table S3. List of qPCR primers

Gene name	Species			Sequence		bp
IAP	Human	Forward	5'	CATACCTGGCTCTGTCCAAGA	3'	21
		Reverse	5'	GTCTGGAAGTTGGCCTTGAC	3'	20
18S rRNA	Human	Forward	5'	CTCAACACGGGAAACCTCAC	3'	20
		Reverse	5'	CGCTCCACCAACTAAGAACG	3'	20
CG5150	Drosophila	Forward	5'	GCTGCTGCCAGGAGTTACATT	3'	21
		Reverse	5'	CTATCGGGGACTATTTTGTCCAC	3'	23
CG10827	Drosophila	Forward	5'	AAGTTGGCTTCTGAACCGAATAA	3'	23
		Reverse	5'	CCCGTGAAGGGGAAGTTTTC	3'	20
Rp49	Drosophila	Forward	5'	AGCATACAGGCCCAAGATCG	3'	20
		Reverse	5'	TGTTGTCGATACCCTTGGGC	3'	20
